# Supplementary material for: Sequencing of distinct wing behaviors during Drosophila courtship
Source: bioRxiv. 2025 Aug 25:2025.08.21.671456. Preprint. [Version 1] doi: 10.1101/2025.08.21.671456 (PMC12407689; doi:10.1101/2025.08.21.671456)
Supplement: Supplement 6 [file NIHPP2025.08.21.671456v1-supplement-6.pdf]

bioRxiv preprint doi: https://doi.org/10.1101/2025.08.21.671456; this version posted August 25, 2025. The copyright holder for this preprint (which was not certified by peer review) is the author/funder, who has granted bioRxiv a license to display the preprint in perpetuity. It is made available under aCC-BY 4.0 International license.

Figure S1

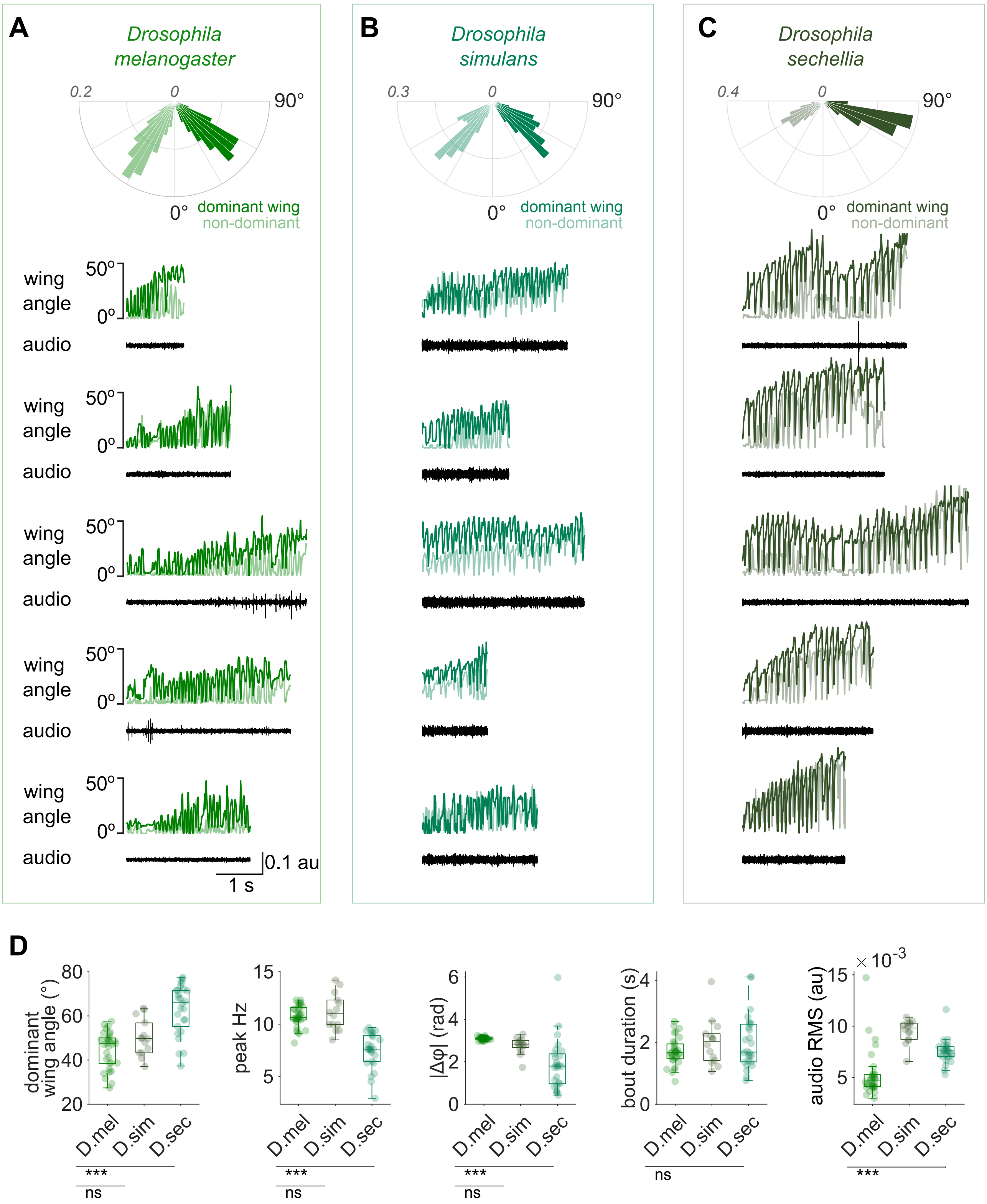

Figure S1 - Related to Figure 1. *Drosophila simulans and Drosophila sechellia* males also produce wing waggling during courtship

A-C. Top: Polar histograms show the distribution of maximum wing extension angles for the dominant (darker shade) and non-dominant (lighter shade) wings during waggling in each species. The dominant wing is defined as the side with the greater mean amplitude over the waggling bout. Bottom: Example

bioRxiv preprint doi: https://doi.org/10.1101/2025.08.21.671456; this version posted August 25, 2025. The copyright holder for this preprint (which was not certified by peer review) is the author/funder, who has granted bioRxiv a license to display the preprint in perpetuity. It is made available under aCC-BY 4.0 International license.

traces from single waggling bouts in each species, showing wing angles and simultaneously recorded microphone audio. All traces share the same scale bar.

D. Species comparison of waggling metrics. Box plots show median ± IQR, whiskers extend to 1.5×IQR, dots mark individual per-fly medians. *D. sechellia* extends its wings wider than either *D. melanogaster* or *D. simulans* (Kruskal–Wallis with Dunn–Šidák post-hoc, ***$p < 0.001$) and has a lower peak waggling frequency (ANOVA with Tukey post-hoc, ***$p < 0.001$). *D. melanogaster* and *D. simulans* exhibit nearly anti-phase wing movements during waggling, whereas *D. sechellia* shows weaker phase locking (ANOVA with Tukey post-hoc, ***$p < 0.001$). Bout durations are comparable across species (ANOVA, $p = 0.20$). Audio amplitude during waggling differs significantly between species (ANOVA, ***$p < 0.001$) but remains much quieter than courtship song (see Figure 1G).

N = 32 flies/1,857 bouts (*D. melanogaster*), 14 flies/174 bouts (*D. simulans*), and 26 flies/528 bouts (*D. sechellia*).

bioRxiv preprint doi: https://doi.org/10.1101/2025.08.21.671456; this version posted August 25, 2025. The copyright holder for this preprint (which was not certified by peer review) is the author/funder, who has granted bioRxiv a license to display the preprint in perpetuity. It is made available under aCC-BY 4.0 International license.

Figure S2

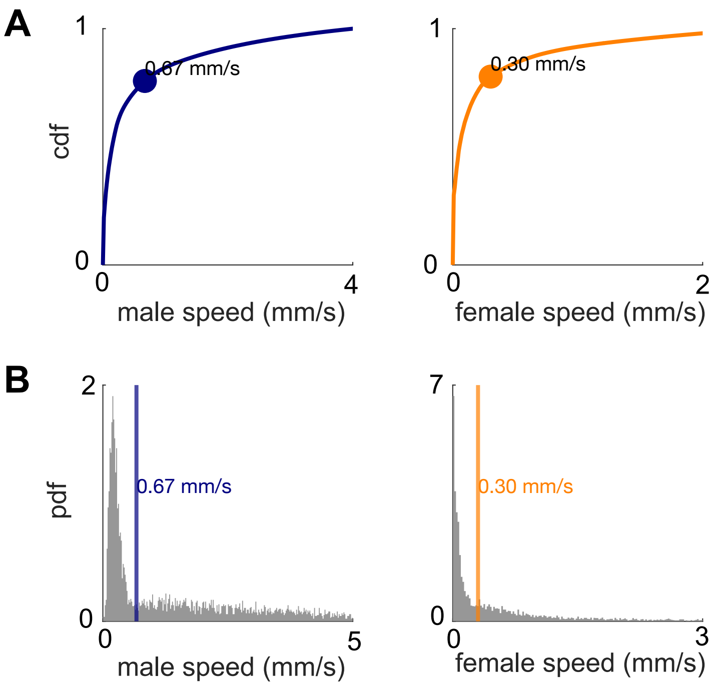

Figure S2 - Related to Figure 2. Defining the “stillness” threshold based on male and female locomotion

A. Cumulative probability distributions (CDFs) of male (navy) and female (orange) speeds across all frames. Dots mark the knee points (inflection points) at 0.67 mm/s for males and 0.30 mm/s for females. These thresholds define the “stillness” threshold, periods when both the male and female remain nearly stationary.

B. Probability density functions (PDFs) of male (left) and female (right) speeds (gray) within waggle and song bouts combined. Vertical lines mark the corresponding stillness thresholds.

bioRxiv preprint doi: https://doi.org/10.1101/2025.08.21.671456; this version posted August 25, 2025. The copyright holder for this preprint (which was not certified by peer review) is the author/funder, who has granted bioRxiv a license to display the preprint in perpetuity. It is made available under aCC-BY 4.0 International license.

Figure S3

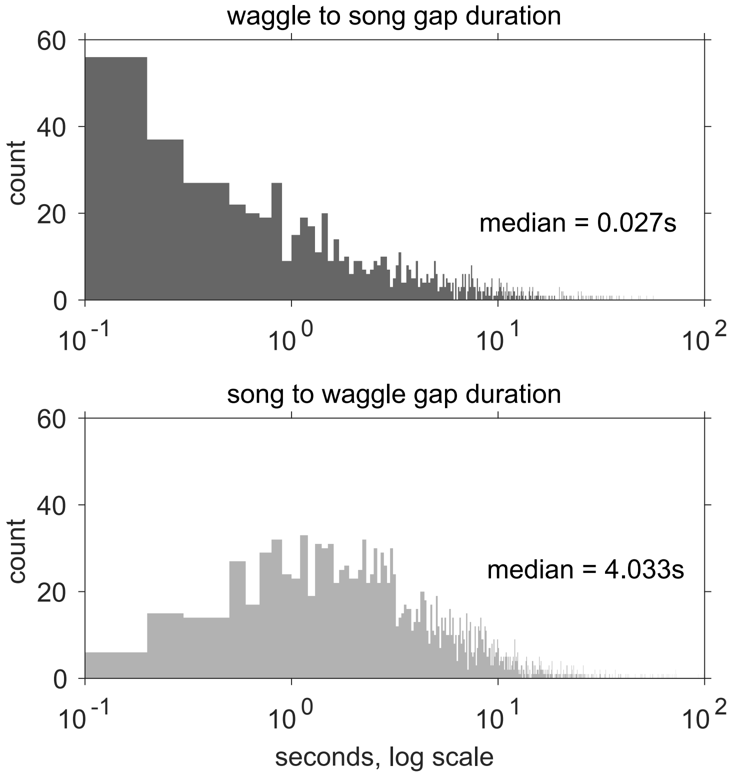

Figure S3 - Related to Figure 3. Waggling is followed by, not preceded by, singing. Top panel: Distribution of gap durations between the end of waggle bouts and the start of the subsequent song bout. Bottom panel: Distribution of gap durations between the start of waggle bouts and the end of the previous song bout.

bioRxiv preprint doi: https://doi.org/10.1101/2025.08.21.671456; this version posted August 25, 2025. The copyright holder for this preprint (which was not certified by peer review) is the author/funder, who has granted bioRxiv a license to display the preprint in perpetuity. It is made available under aCC-BY 4.0 International license.

Figure S4

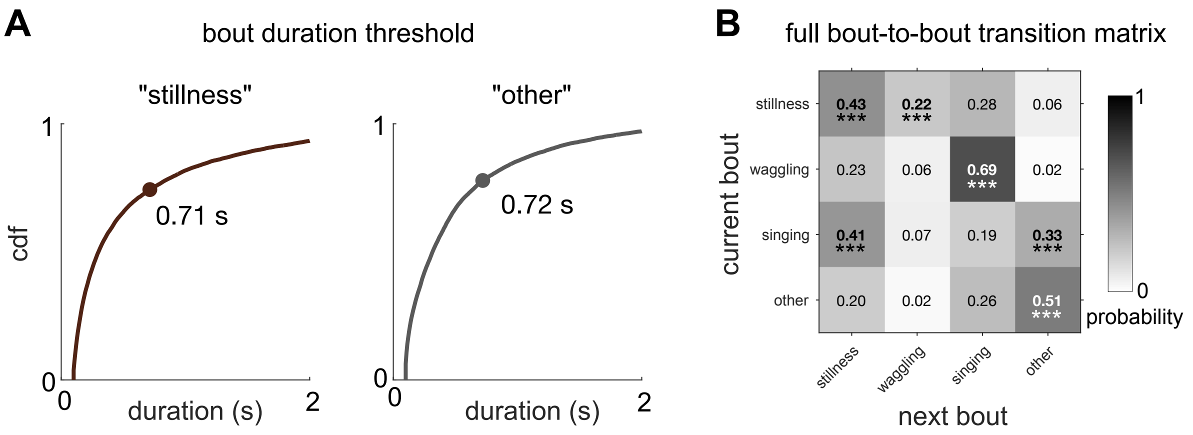

Figure S4 - Related to Figure 4. Bout duration thresholds and full behavioral transition matrix

A. Cumulative probability distributions of durations for "stillness" (left) and "other" (right) states. Dots mark the duration thresholds used to define bouts, identified using the knee-point algorithm (see Methods).

B. Complete four-state bout-to-bout transition probability matrix. Transition probabilities between all behavioral states (stillness, waggling, singing, and other). Statistically significant transitions are highlighted (one-tailed, permutation test, ***p < 0.001). This complements the three-state submatrix shown in Figure 4B.

bioRxiv preprint doi: https://doi.org/10.1101/2025.08.21.671456; this version posted August 25, 2025. The copyright holder for this preprint (which was not certified by peer review) is the author/funder, who has granted bioRxiv a license to display the preprint in perpetuity. It is made available under aCC-BY 4.0 International license.

670 **Figure S5**

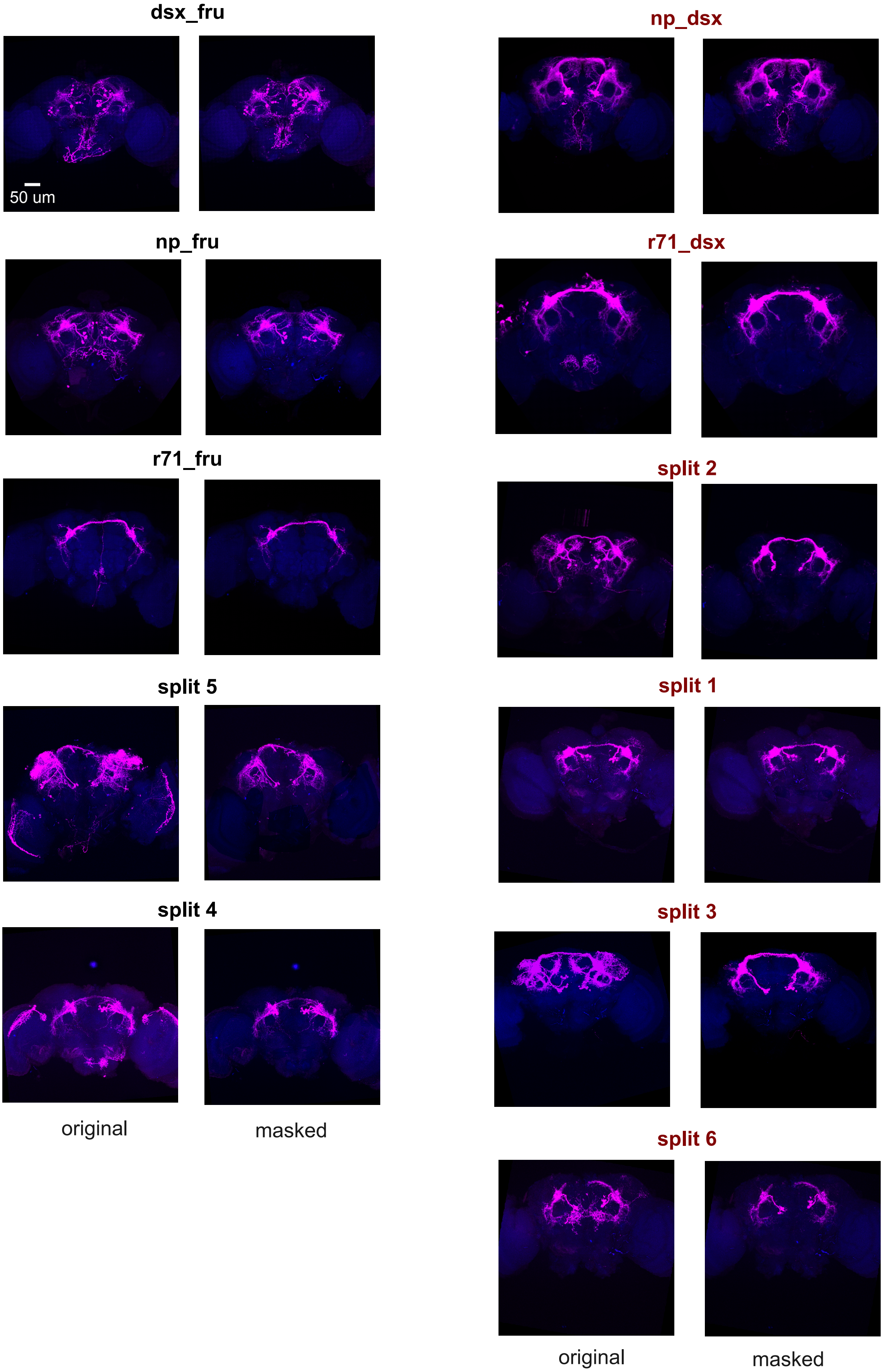

671

672 **Figure S5 - Related to Figure 5. Anatomical expression of P1 neuron subsets.** Maximum
673 intensityprojections of confocal stacks showing brain expression patterns for all 11 genotypes used in

bioRxiv preprint doi: https://doi.org/10.1101/2025.08.21.671456; this version posted August 25, 2025. The copyright holder for this preprint (which was not certified by peer review) is the author/funder, who has granted bioRxiv a license to display the preprint in perpetuity. It is made available under aCC-BY 4.0 International license.

optogenetic experiments (magenta: anti-GFP; blue: neuropil counterstain). Each pair shows original (left) and masked (right) images; masked images have non-P1 expression removed for clarity. While most driver combinations target mostly P1 neurons, some additional neurons are labeled in each line.

bioRxiv preprint doi: https://doi.org/10.1101/2025.08.21.671456; this version posted August 25, 2025. The copyright holder for this preprint (which was not certified by peer review) is the author/funder, who has granted bioRxiv a license to display the preprint in perpetuity. It is made available under aCC-BY 4.0 International license.

677 **Figure S6**

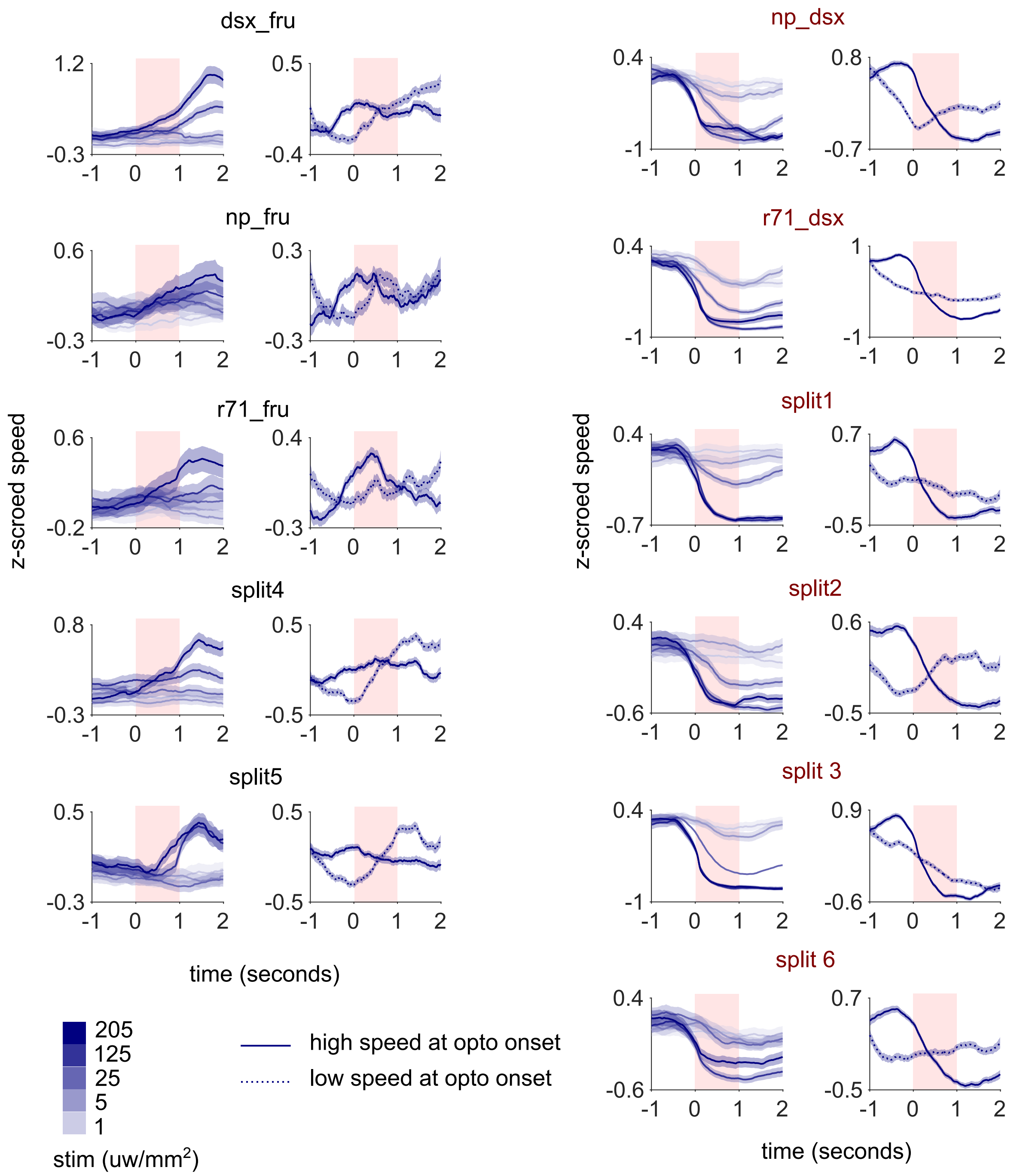

678

679 **Figure S6 - Related to Figure 5. Speed dynamics following optogenetic activation of P1 neuron**
680 **subsets.** Z-scored walking speed aligned to optogenetic onset (red shaded area), sorted by genotype.
681 For each genotype, left panels show responses split by optogenetic stimulus strength (5 levels, light to
682 dark blue); right panels show responses split by initial locomotor state at stimulation onset (high speed:
683 solid lines; low speed: dashed lines). High/low speed groups are determined by median split of speed
684 at optogenetic onset across all stimulus levels.

685 N = 17 (dsx_fru), 15 (np_dsx), 14 (np_fru), 19 (r71_dsx), 15 (r71_fru), 19 (split1/split P1a), 18 (split2),
686 19 (split3), 19 (split4), 19 (split5), 18 (split6) males.

bioRxiv preprint doi: https://doi.org/10.1101/2025.08.21.671456; this version posted August 25, 2025. The copyright holder for this preprint (which was not certified by peer review) is the author/funder, who has granted bioRxiv a license to display the preprint in perpetuity. It is made available under aCC-BY 4.0 International license.

Figure S7

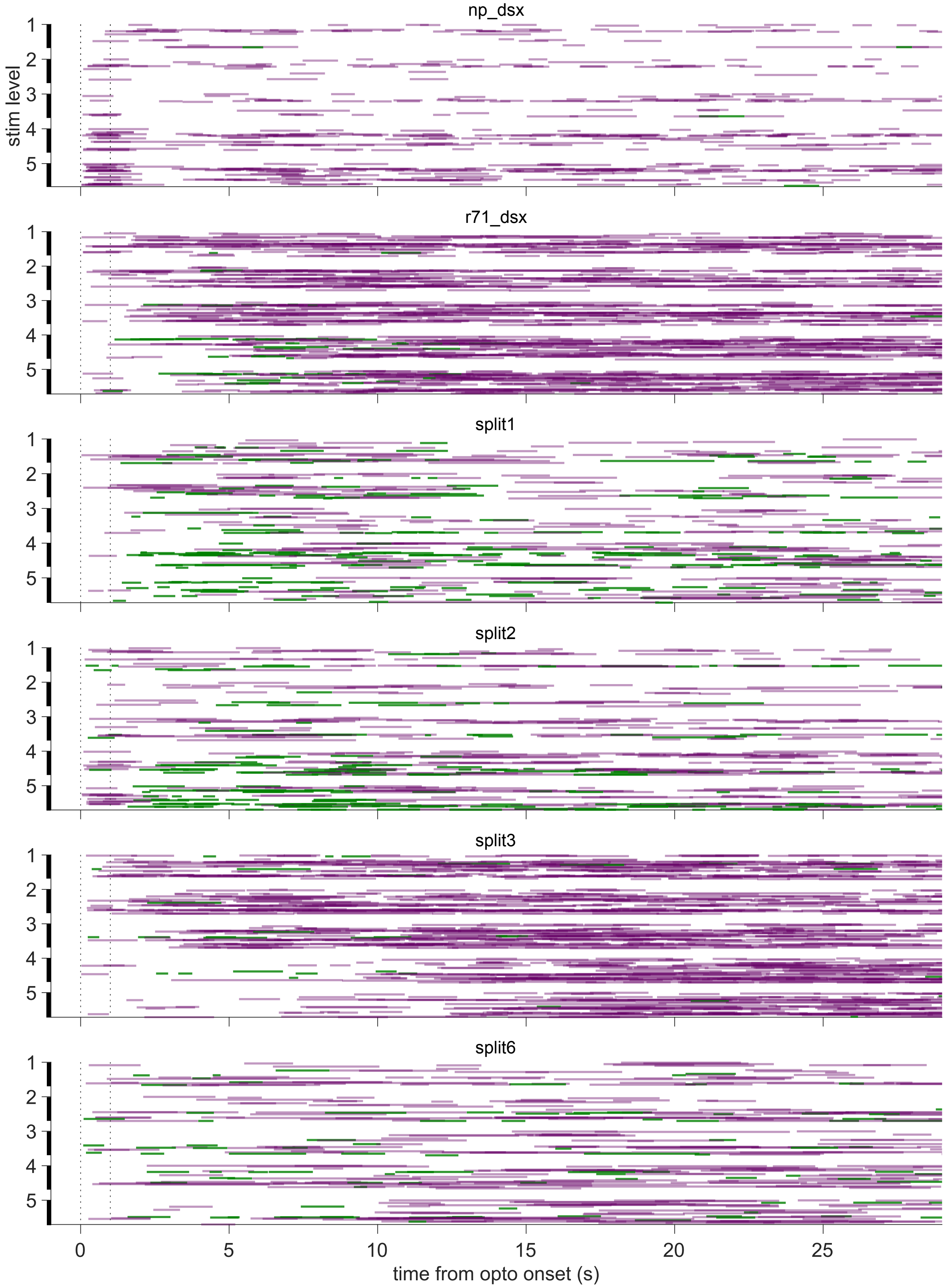

Figure S7 - Related to Figure 5. Raster plots of optogenetically evoked waggling and singing. Behavioral responses for each effective genotype following optogenetic stimulation. Each row

bioRxiv preprint doi: https://doi.org/10.1101/2025.08.21.671456; this version posted August 25, 2025. The copyright holder for this preprint (which was not certified by peer review) is the author/funder, who has granted bioRxiv a license to display the preprint in perpetuity. It is made available under aCC-BY 4.0 International license.

represents a single trial, with horizontal bars indicating detected waggle (green) and song (purple) bouts. Dashed lines represent onset and offset of optogenetic stimulation. Trials are grouped by stimulation strength (levels 1-5: 1, 5, 25, 125, 205 $\mu W/mm^2$).

N = 15 (np_dsx), 19 (r71_dsx), 19 (split1/split P1a), 18 (split2), 19 (split3), 18 (split6) males.

bioRxiv preprint doi: https://doi.org/10.1101/2025.08.21.671456; this version posted August 25, 2025. The copyright holder for this preprint (which was not certified by peer review) is the author/funder, who has granted bioRxiv a license to display the preprint in perpetuity. It is made available under aCC-BY 4.0 International license.

Figure S8

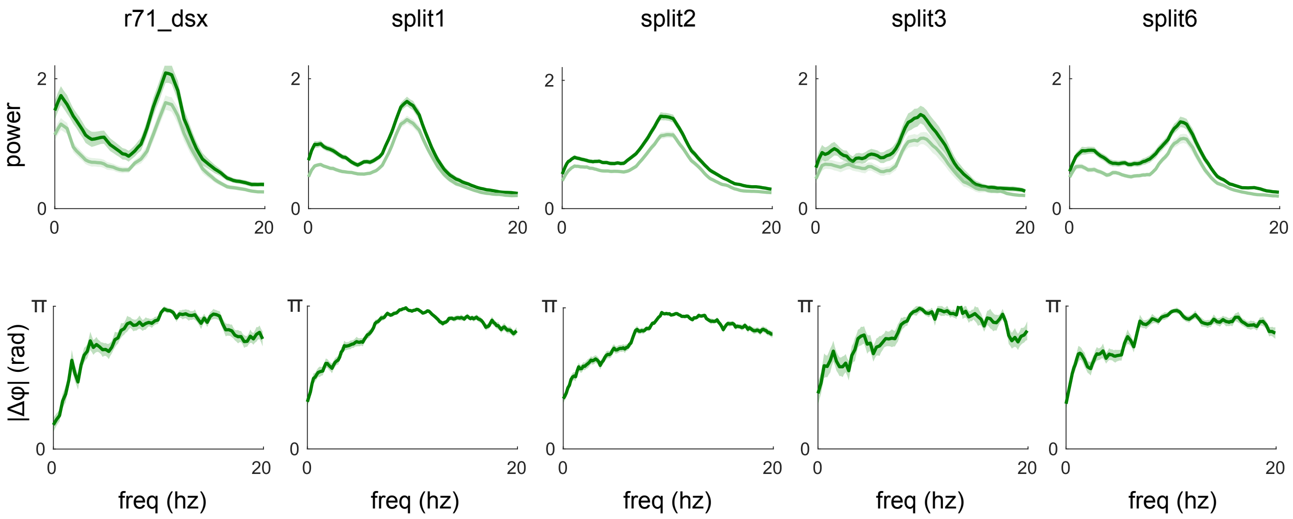

Figure S8 - Related to Figure 5. Wing movement characteristics of optogenetically evoked waggling. Power spectra (top) and absolute inter-wing phase difference |Δφ| (bottom) during optogenetically evoked waggling bouts for each effective genotype (mean ± SEM).

N = 19 (r71_dsx), 19 (split1/split P1a), 18 (split2), 19 (split3), 18 (split6) males.

bioRxiv preprint doi: https://doi.org/10.1101/2025.08.21.671456; this version posted August 25, 2025. The copyright holder for this preprint (which was not certified by peer review) is the author/funder, who has granted bioRxiv a license to display the preprint in perpetuity. It is made available under aCC-BY 4.0 International license.

Table S1

abbreviation used in this manuscript	genotype	reference
split1	UAS-CsChrimson.mVenus; R15A01.AD/+; R71G01.DBD/+	Hoopfer et al.[1], as P1^{a}
split2	UAS-CsChrimson.mVenus; R17D06.AD/+; R71G01.DBD/+	Zhang et al.[2], as P1^{c}
split3	UAS-CsChrimson.mVenus; R22D03.AD/+; R71G01.DBD/+	
split4	UAS-CsChrimson.mVenus; R15A01.AD/+; R17D06.DBD/+	
split5	UAS-CsChrimson.mVenus; R22D03.AD/+; R17D06.DBD/+	Zhang et al.[2], same combination as P1^{d}
split6	UAS-CsChrimson.mVenus; R15A01.AD/+; R22D03.DBD/+	Zhang et al.[2], as P1^{b}
dsx_fru	;UAS>stop> CsChrimson.mVenus/+; dsx^{Gal4}/ Fru^{FLP}	Ishii et al.[3],similar to dsx^{GAL4}, UAS > stop > CsChrimson, fru^{FLP}
r71_fru	;UAS>stop> CsChrimson.mVenus/+ ; Fru^{FLP}/R71G01	Coleman et al.[4], similar to w; 71G01-p65.AD/+;UAS-CsChrimson, fru-DBD/+ +
r71_dsx	+/UAS>stop> CsChrimson.mVenus; dsx-LexA, 8xLexAop2-FLP/R71G01	Coleman et al.[4], similar to w; 71G01-p65.AD/UAS-CsChrimson; dsx-DBD/+
np_fru	;NP2631/UAS>stop> CsChrimson.mVenus; Fru^{FLP}/+	
np_dsx	NP2631/UAS>stop> CsChrimson.mVenus; dsx-LexA, 8xLexAop2-FLP/+	Ishii et al.[3], similar to NP2631, UAS > stop > CsChrimson, dsx^{FLP}

Table S1- Related to Figure 5. Genotypes of P1 driver lines used for optogenetic experiments.

Supplementary References:

1. Hoopfer ED, Jung Y, Inagaki HK, Rubin GM, Anderson DJ. P1 interneurons promote a persistent internal state that enhances inter-male aggression in Drosophila. Elife. 2015;4. doi:10.7554/eLife.11346
2. Zhang W, Guo C, Chen D, Peng Q, Pan Y. Hierarchical Control of Drosophila Sleep, Courtship, and Feeding Behaviors by Male-Specific P1 Neurons. Neurosci Bull. 2018;34: 1105–1110.
3. Ishii K, Wohl M, DeSouza A, Asahina K. Sex-determining genes distinctly regulate courtship capability and target preference via sexually dimorphic neurons. Elife. 2020;9. doi:10.7554/eLife.52701
4. Coleman RT, Morantte I, Koreman GT, Cheng ML, Ding Y, Ruta V. A modular circuit coordinates the diversification of courtship strategies. Nature. 2024;635: 142–150.
